# Targeting the epigenetic regulation of ferroptosis: a potential therapeutic approach for sepsis-associated acute kidney injury

**DOI:** 10.1186/s13148-025-01861-9

**Published:** 2025-04-06

**Authors:** Yuhang Yang, Xinqi Deng, Wenyuan Li, Yan Leng, Yonghong Xiong, Bihan Wang, Siyuan Gong, Yunhao Wang, Baichuan Yang, Wei Li

**Affiliations:** Department of Anesthesiology, Renmin Hospital of Wuhan University, Wuhan, 430060 Hubei China

**Keywords:** Acute kidney injury, Sepsis, Ferroptosis, Epigenetic regulation

## Abstract

Sepsis is a syndrome of organ dysfunction caused by the invasion of pathogenic microorganisms. In clinical practice, patients with sepsis are prone to concurrent acute kidney injury, which has high morbidity and mortality rates. Thus, understanding the pathogenesis of sepsis-associated acute kidney injury is of significant clinical importance. Ferroptosis is an iron-dependent programmed cell death pathway, which is proved to play a critical role in the process of sepsis-associated acute kidney injury through various mechanisms. Epigenetic regulation modulates the content and function of nucleic acids and proteins within cells through various modifications. Its impact on ferroptosis has garnered increasing attention; however, the role of epigenetic regulation targeting ferroptosis in sepsis-associated acute kidney injury has not been fully elucidated. Growing evidence suggests that epigenetic regulation can modulate ferroptosis through complex pathway networks, thereby affecting the development and prognosis of sepsis-associated acute kidney injury. This paper summarizes the impact of ferroptosis on sepsis-associated acute kidney injury and the regulatory mechanisms of epigenetic regulation on ferroptosis, providing new insights for the targeted therapy of sepsis-associated acute kidney injury.

## Introduction

**Fig. 1:**
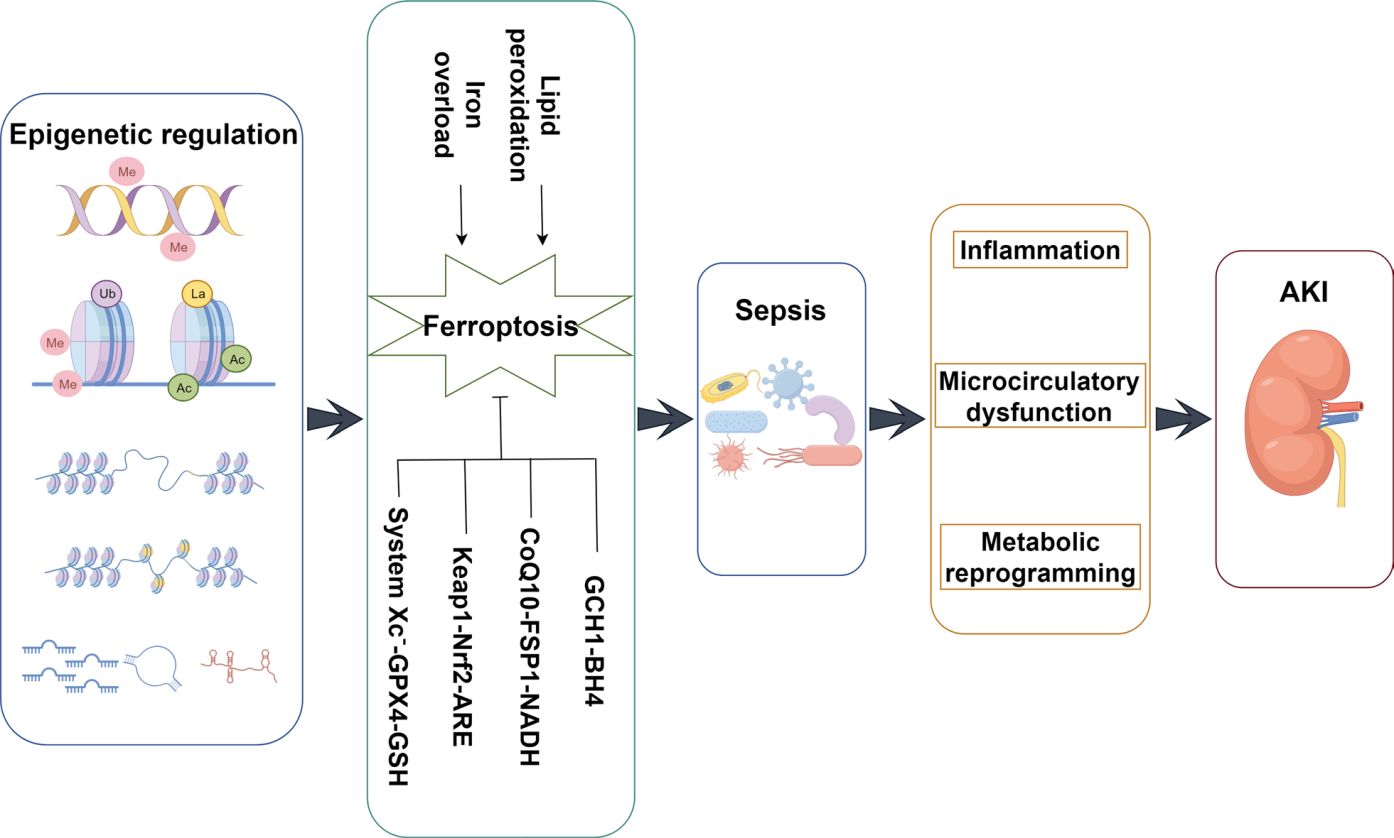
The logical framework of this paper (By Figdraw)

Acute kidney injury (AKI) is a clinical syndrome characterized by a rapid decline in kidney function due to various causes. It is accompanied by a sudden decrease in glomerular filtration rate and mainly manifests as elevated serum creatinine or oliguria, and even anuria in severe cases, which can progress to renal failure or even death [1]. AKI can be caused by multiple factors, with sepsis being one of the primary causes. Studies show that approximately 60% of sepsis patients develop AKI along with related adverse clinical symptoms [2]. With the continuous updating of definitions, sepsis is now defined as a life-threatening organ dysfunction syndrome caused by a dysregulated host response to infection [3]. Ferroptosis is an iron-dependent form of programmed cell death which is characterized by intracellular iron overload and lipid peroxidation during the cell death process [4]. In recent years, numerous experiments have shown that ferroptosis involves a precise regulatory network and is considered as an important aspect of the development, treatment, and early prevention of sepsis-associated AKI. Epigenetic regulation controls chromatin structure and gene expression by writing, reading and erasing the covalent modification of DNA and histones, without altering the DNA sequence [5]. Increasing evidence suggests that epigenetic regulation is involved in the occurrence and regulation of ferroptosis in cells, playing a crucial role in the development and prognosis of sepsis-associated AKI. This paper analyzes the relationship between sepsis-associated AKI, ferroptosis and epigenetic regulation, focusing on the mechanisms of ferroptosis in sepsis-associated AKI and the regulatory role of epigenetic regulation on ferroptosis, providing significant reference value for the clinical treatment of sepsis-associated AKI (Fig. 1).

## Overview of sepsis-associated AKI

Sepsis induces AKI through various mechanisms, with the three most important being inflammation, microcirculatory dysfunction, and metabolic reprogramming [6]. Additionally, dysregulated immune response, hemodynamic changes, and mitochondrial dysfunction have also been shown to play significant roles.

### Inflammation

After bacterial infection, pathogenic microorganisms promote the release of inflammatory mediators from both the bacteria and host immune cells, known as pathogen-associated molecular patterns and damage-associated molecular patterns (DAMPs). These mediators can bind to pattern recognition receptor (PRR), initiating downstream signaling cascades and enhancing the synthesis and release of pro-inflammatory molecules [7].

Toll-like receptor (TLR) is a type of PRR expressed on the surface of renal tubular epithelial cells. In response to infection, DAMPs and pathogen-associated molecular patterns bind to TLR and activate nuclear factor kappa-B, upregulating the expression of inflammatory cytokine genes, which leads to the generation of ROS, oxidative stress, and mitochondrial damage, exacerbating injury to renal tubular epithelial cells [8, 9]. Additionally, TLR4 not only triggers MyD88-dependent signaling to induce NF-κB activation, but also activates TRIF-dependent signaling through endocytosis, promoting the production of type I interferon [10, 11]. The NOD-like receptor protein 3 (NLRP3) inflammasome is another type of PRR that plays a crucial role in the inflammatory cascade of sepsis-associated AKI. The NLRP3 inflammasome can activate caspase-1, promoting the maturation of IL-1β and IL-18, thus exacerbating the inflammatory response [12]. In a sepsis mouse model, knocking out NLRP3 reduced the levels of caspase-1 and interleukins in the kidney and alleviated renal injury, confirming the role of the NLRP3 inflammasome in inflammation development [4, 13]. In summary, inflammation is a critical factor in sepsis-associated AKI.

### Microcirculatory dysfunction

Reduced renal blood flow leading to acute tubular necrosis was once regarded as the primary cause of sepsis-associated AKI. However, recent studies have shown that renal blood flow during sepsis can be normal or even increased despite ongoing tissue hypoxia, indicating that sepsis mainly affects kidney through microcirculatory dysfunction [14].

During sepsis, the contraction of the afferent arterioles and the dilation of the efferent arterioles reduce the hydrostatic pressure of the glomerulus. Concurrently, the contraction of afferent arterioles causes intrarenal shunting through the extraglomerular capillaries, bypassing the glomerulus entirely, which leads to a reduced glomerular filtration rate and promotes the formation of microthrombi [15]. Additionally, coagulation function can also change in patients with sepsis, which leads to increased rolling and adhesion of leukocyte and platelet, decreased blood flow velocity, formation of microthrombus formation, and ultimately affects microvascular flow [16].

Studies have demonstrated that improving renal microcirculation in sepsis-associated AKI rats can suppress renal pathological alterations and enhance renal function [17]. After the occurrence of microcirculatory dysfunction and septic shock, reducing renal capillary permeability to restore renal microcirculation can also improve renal function and protect the kidney [18]. In conclusion, microcirculatory dysfunction contributes to the development of sepsis-associated AKI, which highlights the importance of improving renal microcirculation in its treatment.

### Metabolic reprogramming

Metabolic reprogramming is a mechanism by which cells alter their metabolic patterns to meet energy demands, promoting cell proliferation and growth, and is most commonly observed in cancer cells [19]. Under physiological conditions, renal cells utilize aerobic respiration to produce ATP. However, during sepsis, distinct alterations occur in the metabolic sequence of renal tubular epithelial cells.

During the early metabolic response, the metabolism of renal tubular epithelial cells is dominated by aerobic glycolysis, while oxidative phosphorylation (OXPHOS) is inhibited, leading to the activation of cellular inflammation [20]. However, during the adaptive catabolic stage, the metabolism of renal tubular epithelial cells shifts from aerobic glycolysis to OXPHOS, resulting in a decrease in cellular inflammation [21]. Related experiments have shown that upregulating the expression of pyruvate kinase M2 in cells can promote aerobic glycolysis, leading to the production of high concentrations of lactate, which promotes the acetylation and release of high mobility group protein 1, thereby enhancing cellular inflammatory responses [22]. The activation of Adenosine 5 ‘-monophosphate (AMP)-activated protein kinase (AMPK), on the other hand, improves OXPHOS and mitochondrial function and maintains metabolic adaptability and flexibility [23]. Furthermore, as a key intermediate in glycolysis, lactate production is stimulated by increased pyruvate levels during sepsis [24]. This process may be closely associated with adrenaline-driven glycolysis and Na⁺/K⁺-ATPase pump activation [25]. Experimental studies indicate that lowering lactate levels can mitigate the progression of sepsis-associated AKI [26], suggesting that targeting lactate could be a potential therapeutic strategy for sepsis-associated AKI.

During sepsis, not only does glucose metabolism change, but lipid and amino acid metabolism also undergo alterations [27, 28]. However, relevant research is limited, which could serve as a direction for future studies. In summary, the early phase of metabolic reprogramming promotes cellular inflammatory responses, mitochondrial autophagy, and the production of lower levels of ATP, but the limited ATP is redirected to essential functions, reducing ROS production and protecting the kidneys from further damage; the adaptive catabolic phase inhibits cellular inflammatory responses and improves mitochondrial function, increasing survival rates [29].

## The mechanism of ferroptosis

**Fig. 2:**
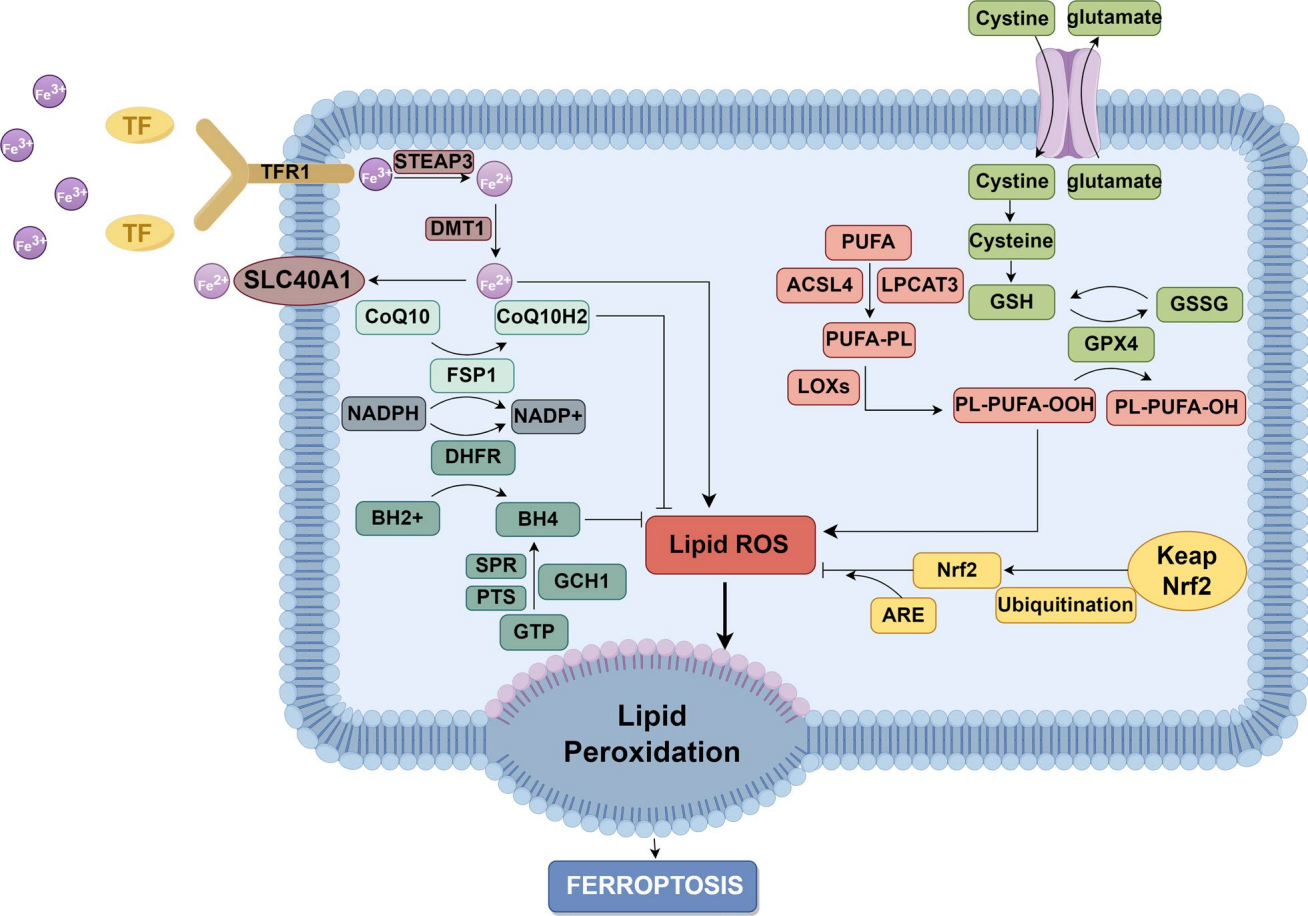
Schematic diagram of ferroptosis. Iron overload and lipid peroxidation can promote the occurrence of ferroptosis, while System Xc.^−^-GPX4-GSH, Keap1-Nrf2-ARE, CoQ10-FSP1-NADH, and GCH1-BH4 pathways can inhibit the occurrence of ferroptosis. Transferrin receptor 1 (TFR1); solute carrier family 40 member 1 (SLC40A1); STEAP3 metalloreductase (STEAP3); Divalent Metal Transporter 1 (DMT1); Coenzyme Q10 (CoQ10); Ferroptosis suppressor protein 1 (FSP1); Dihydrofolate reductase (DHFR); Tetrahydrobiopterin (BH4); GTP cyclohydrolase 1 (GCH1); Pyruvoyl tetrahydrobiopterin synthase (PTS); Sepiapterin Reductase (SPR); glutathione (GSH); Oxidized glutathione (GSSG); glutathione peroxidase 4 (GPX4); Polyunsaturated fatty acid (PUFA); Lysophosphatidylcholine Acyltransferase 3 (LPCAT3); Acyl-CoA synthetase long-chain family member 4 (ACSL4); Lipoxygenases (LOXs); Nuclear factor erythroid-2-related factor 2 (Nrf2); Kelch-like ECH-associated protein 1 (Keap1); antioxidant response element (ARE) (By Figdraw)

Ferroptosis was first proposed by Dixon in 2012 and is described as a unique form of iron-dependent non-apoptotic cell death, distinct from apoptosis, necrosis, and autophagy in terms of morphology, biochemistry, and genetics [4]. Ferroptosis is characterized by dysregulated iron metabolism, lipid peroxidation, and the involvement of specific regulatory pathways (Fig. 2).

### Iron in ferroptosis

In the human circulatory system, Fe^3+^ binds to transferrin and enters cells through transferrin receptor 1 (TFR1). Iron in Fe^3+^ form is deoxidized to iron in Fe^2+^ which was then released into the cytoplasmic labile iron pool. Excess iron is stored in ferritin or exported from cells via ferroportin [30]. When iron overload occurs, excess Fe^2+^ in the labile iron pool generates highly reactive hydroxyl radicals via the Fenton reaction (Fe^2+^ + H_2_O_2_ → Fe^3+^ + •OH + OH^−^), which can react with polyunsaturated fatty acid (PUFA) in cell membranes, producing large amounts of ROS, which promotes ferroptosis in cells [31].

### Lipid peroxidation

Lipids are the main component of cell membranes and play a crucial role in maintaining cellular homeostasis. Due to the high content of unsaturated double bonds in the PUFA of cell membranes, they become the primary targets of ROS. Under the catalysis of Acyl-CoA synthetase long-chain family member 4 (ACSL4) and Lysophosphatidylcholine Acyltransferase 3, PUFAs are synthesized and inserted into membrane phospholipids to form PUFA-PL complexes, which are then oxidized by lipoxygenases to generate toxic PUFA-PL-OOH, thereby activating ferroptosis [32]. In recent years, studies have found that the endoplasmic reticulum membrane is a key site for lipid peroxidation. During ferroptosis, lipid peroxidation initially accumulates in the endoplasmic reticulum membrane and subsequently in the plasma membrane [33], which have significant implications for the design of targeted therapies.

### Regulation of ferroptosis

System Xc^−^ is a heterodimer composed of a light chain and a heavy chain, with solute carrier family 7 member 11 (SLC7A11) being the key component regulating its activity [34]. System Xc^−^ transports cystine into cells, which is involved in the synthesize of glutathione (GSH) to provide electrons in the reaction catalyzed by glutathione peroxidase 4 (GPX4), reducing toxic phospholipid hydroperoxides to non-toxic phospholipid alcohols, thus mitigating lipid peroxidation and inhibiting ferroptosis [35].

Nuclear factor erythroid-2-related factor 2 (Nrf2) is a transcription factor that binds to nuclear factors and regulates the expression of cellular antioxidant genes. In the cytoplasm, Kelch-like ECH-associated protein 1 (Keap1) complexes with Nrf2. Under oxidative stress, Keap1 is degraded, while Nrf2 is released and enters the nucleus to bind with antioxidant response element (ARE), activating the transcription of antioxidant genes [36]. System Xc^−^ and GPX4 are two of the most critical targets that have been shown to be regulated by Nrf2, and their activation can inhibit ferroptosis [37].

Coenzyme Q10 (CoQ10), also known as ubiquinone, plays a crucial role in the mitochondrial electron transport chain. Ferroptosis suppressor protein 1 (FSP1) reduces CoQ10 to ubiquinol, effectively reducing lipid peroxidation and preventing ferroptosis [38].

Tetrahydrobiopterin (BH4) maintains cellular redox stability which has recently been found to regulate ferroptosis. GTP cyclohydrolase 1 (GCH1) promotes the reduction of GTP to BH4, reducing intracellular ROS and inhibits ferroptosis [39].

## The relationship between sepsis and iron homeostasis

Iron is an essential trace element for most organisms, including bacteria. Almost all bacteria require iron as a cofactor for enzymes that catalyze redox reactions involved in fundamental cellular processes. During sepsis, the host competes with pathogens for iron. The liver synthesizes and releases hepcidin, which induces the degradation of ferroportin to prevent the loss of iron from cells [40]. The primary mechanism by which bacteria acquire this trace element from the environment is secreting siderophores, which are secondary metabolites that form soluble Fe^3+^ complexes to obtain iron from environmental stores and then actively transport it into the bacteria through specific receptors [41]. Studies have shown that iron is closely related to the pathogenicity of certain microorganisms, such as Escherichia coli and Klebsiella pneumoniae. Although the impact of iron varies among different microorganisms, generally, an iron-rich environment is more conducive to microbial growth. Randomized observational studies have also found that increased iron levels promote the development of sepsis [42].

Recent studies have demonstrated a close relationship between iron, inflammation, metabolic reprogramming, and microcirculatory dysfunction. Allison et al. demonstrated that cellular iron overload could act as a switch driving cells into inflammation and metabolic reprogramming [43]. Another study indicated that iron homeostasis disorders are closely related to an increase in the labile iron pool, promoting the production of ROS, leading to vascular endothelial injury and accelerating vascular aging. Inflammation caused by iron homeostasis disorders can also lead to changes in both microvascular and macrovascular structures. This suggests that iron homeostasis is closely related to microcirculatory dysfunction in sepsis-associated AKI [44].

Hepcidin can reduce serum iron, decrease the cytotoxic environment throughout the body, and mitigate inflammation induced by sepsis, thereby alleviating AKI [45]. During sepsis, hepcidin secretion increases, leading to iron sequestration in macrophages, causing iron retention in cells and effectively reducing plasma iron. This helps limit the availability of iron to pathogens, inhibiting their proliferation and protecting the body [46]. Increasing experimental evidence demonstrates that hepcidin plays a significant protective role in sepsis-associated AKI through its anti-inflammatory and antimicrobial properties [45].

In summary, iron overload promotes the occurrence and development of sepsis- associated AKI, whereas hepcidin, a regulatory factor that increases reactively during sepsis, can inhibit the proliferation of pathogens by reducing serum iron levels, thereby mitigating kidney damage.

## The impact of ferroptosis on sepsis-associated AKI

There is a close relationship between iron homeostasis and sepsis, and ferroptosis has also been confirmed as an important regulatory link in sepsis- associated AKI. The development of sepsis-associated AKI involves many mechanisms, mainly including inflammation, microcirculatory dysfunction, and metabolic reprogramming. Understanding the role of ferroptosis in these mechanisms is crucial for clarifying the role of ferroptosis in sepsis- associated AKI.

### Ferroptosis and inflammation

**Fig. 3:**
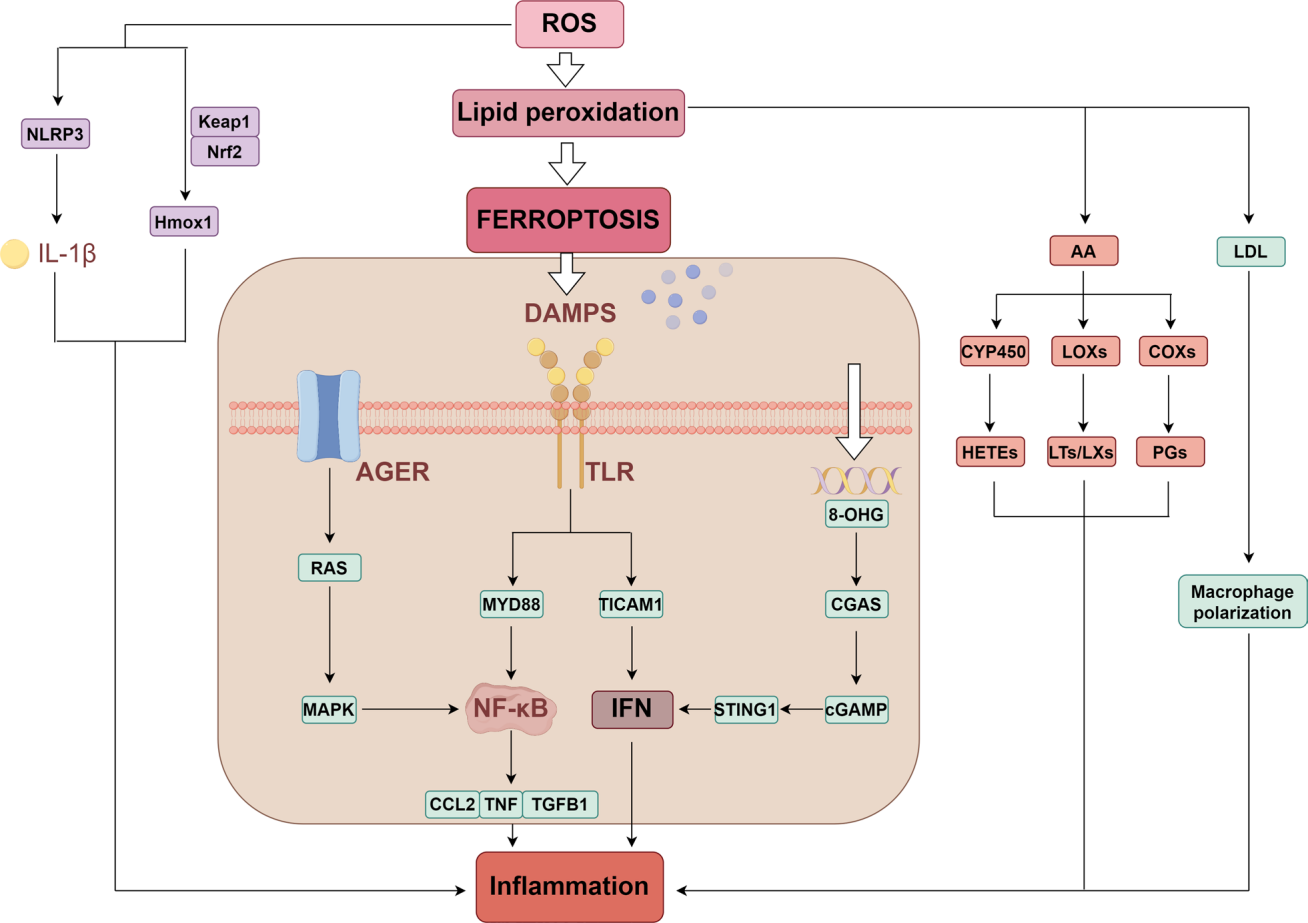
The regulatory mechanism of ferroptosis on inflammation. Ferroptosis can promote cell damage, causing stressed cells to release DAMPs. DAMPs can promote inflammation through AGER, TLR4, and CGAS. In the process of lipid peroxidation, AA is released from phospholipids and metabolized by COXs, LOX, and CYP450 into inflammatory mediators. Furthermore, LDL released during lipid peroxidation can promote macrophage polarization and enhance inflammation. ROS produced during ferroptosis can promote the activation of the NLRP3 inflammasome and the Keap1/Nrf2/Hmox1 pathway, which also play significant roles in inflammation. NOD-like receptor protein 3 (NLRP3); Heme oxygenase 1 (Hmox1); Advanced glycosylation end-product specific receptor (AGER); mitogen-activated protein kinase (MAPK); Myeloid Differentiation Factor 88 (MYD88); Toll Like Receptor Adaptor Molecule 1 (TICAM1); 8-hydroxyguanine (8-OHG); cyclic GMP-AMP synthase (CGAS); Cyclic GMP-AMP (cGAMP); Stimulator of interferon response cGAMP interactor 1 (STING1); C–C motif chemokine ligand 2 (CCL2); transforming growth factor beta 1 (TGFB1); arachidonic acid (AA); cytochrome P450 (CYP450); Lipoxygenases (LOXs); Cyclooxygenases (COXs); Hydroxyeicosatetraenoic acids (HETEs); Low density lipoprotein (LDL) (By Figdraw)

Inflammation plays a crucial role in the process of sepsis-associated AKI. Research has shown that ferroptosis is accompanied by the release of inflammatory molecules [47]. In addition to mechanisms such as apoptosis, extracellular traps, secretory lysosomes, and exosomes, ferroptosis can also promote the release of DAMPs [48]. DAMP signals emitted by ferroptosis can be integrated by certain PRRs, such as TLR4 and advanced glycosylation end-product specific receptor (AGER) signaling, thereby triggering inflammation [49]. Furthermore, ROS, arachidonic acid, PUFAs, low-density lipoprotein, and mitochondria also play important roles in the process of ferroptosis promoting inflammation (Fig. 3) [50].

Currently, many studies have demonstrated that inhibiting ferroptosis can alleviate inflammation and reduce the damage caused by sepsis. Melanin nanoparticles have iron-chelating properties, which can inhibit the ferroptosis pathway by reducing iron accumulation. Research indicates that melanin nanoparticles alleviate sepsis-induced damage in mice by inhibiting ferroptosis and inflammation [51]. Another study indicated that Maresin conjugates in tissue regeneration 1 can inhibit ferroptosis by activating Nrf2 signaling, actively regulating the resolution of inflammation and protecting the kidneys [52].

In recent years, research into the regulatory role of inflammation in ferroptosis has expanded. Chen et al. summarized five classic inflammatory pathways and elucidated their roles in ferroptosis [53]. Another study found that knocking out the NLRP3 inflammasome can inhibit renal ferroptosis in mice and reduce renal inflammation, indicating that NLRP3 can regulate ferroptosis in sepsis-associated AKI [54].

The above discussion suggests an interaction between ferroptosis and inflammation, where inhibiting ferroptosis reduces inflammation and thereby alleviates sepsis-associated AKI, offering a new therapeutic approach for the treatment of sepsis-associated AKI.

### Ferroptosis and microcirculatory dysfunction

After invading the human body, pathogenic microorganisms cause microcirculatory dysfunction in the kidneys, promoting the development of AKI. Microcirculatory dysfunction is not only related to factors such as inflammation and amino acid metabolism, but also connected with lipid mediators [55]. One of the key characteristics of ferroptosis is lipid peroxidation, suggesting a potential relationship between ferroptosis and microcirculatory dysfunction. Non-transferrin-bound iron is a form of iron commonly found in various diseases characterized by iron overload, readily undergoing the Fenton reaction to induce the production of ROS. ROS accelerate vascular aging through the peroxidation of lipids and proteins in cell membranes and direct effects on macrophages. Additionally, non-transferrin-bound iron contributes to the uncoupling of endothelial nitric oxide synthase, which reduces nitric oxide availability, leads to impaired endothelium-dependent vasodilation, and subsequently affects microcirculation function [44].

A study on atherosclerosis and ferroptosis indicated that inhibiting ferroptosis with ferrostatin-1 can reduce lipid peroxidation and endothelial dysfunction, thereby alleviating atherosclerosis [56]. Isorhapontigenin, an antioxidant drug, can inhibit mitochondria-related ferroptosis, thereby reducing microvascular damage in the body [57]. These experiments suggest that inhibiting ferroptosis can protect the microvasculature, providing an alternative approach for the treatment of sepsis-associated AKI.

### Ferroptosis and metabolic reprogramming

Increasing research indicates a dynamic interaction between ferroptosis and glycolysis, lipid metabolism, and amino acid metabolism, suggesting that the link between ferroptosis and metabolic reprogramming may have significant implications for sepsis-associated AKI [58]. A study related to Nrf2 shows that enhancing the nuclear accumulation of Nrf2 to a level can promote metabolic activities that support cell proliferation, enhancing the metabolic reprogramming triggered by proliferation signals, indicating a potential connection between ferroptosis and metabolic reprogramming [59].

During the early response to sepsis, cellular metabolism is mainly aerobic glycolysis, leading to substantial lactate accumulation. A study indicates that high concentrations of lactate can promote the production of ATP and inactivate AMPK, increasing the generation of monounsaturated fatty acids, thereby enhancing cellular resistance to ferroptosis [60]. In addition, lactate can inhibit ACSL4 through other pathways and reduce the production of PUFAs, collaboratively resisting ferroptosis [61]. During the adaptive catabolic phase, cellular metabolism shifts from aerobic glycolysis to OXPHOS. Although OXPHOS produces more ATP, it also generates more ROS, causing ferroptosis more likely to occur during OXPHOS metabolism. Compared to normal cells, cells dependent on OXPHOS are more susceptible to glutathione depletion and ferroptosis [62]. This suggests that metabolic reprogramming during sepsis can inhibit ferroptosis, thereby protecting cells and delaying the development of sepsis-associated AKI.

## The role of epigenetic regulation targeting ferroptosis in sepsis-associated AKI

**Fig. 4:**
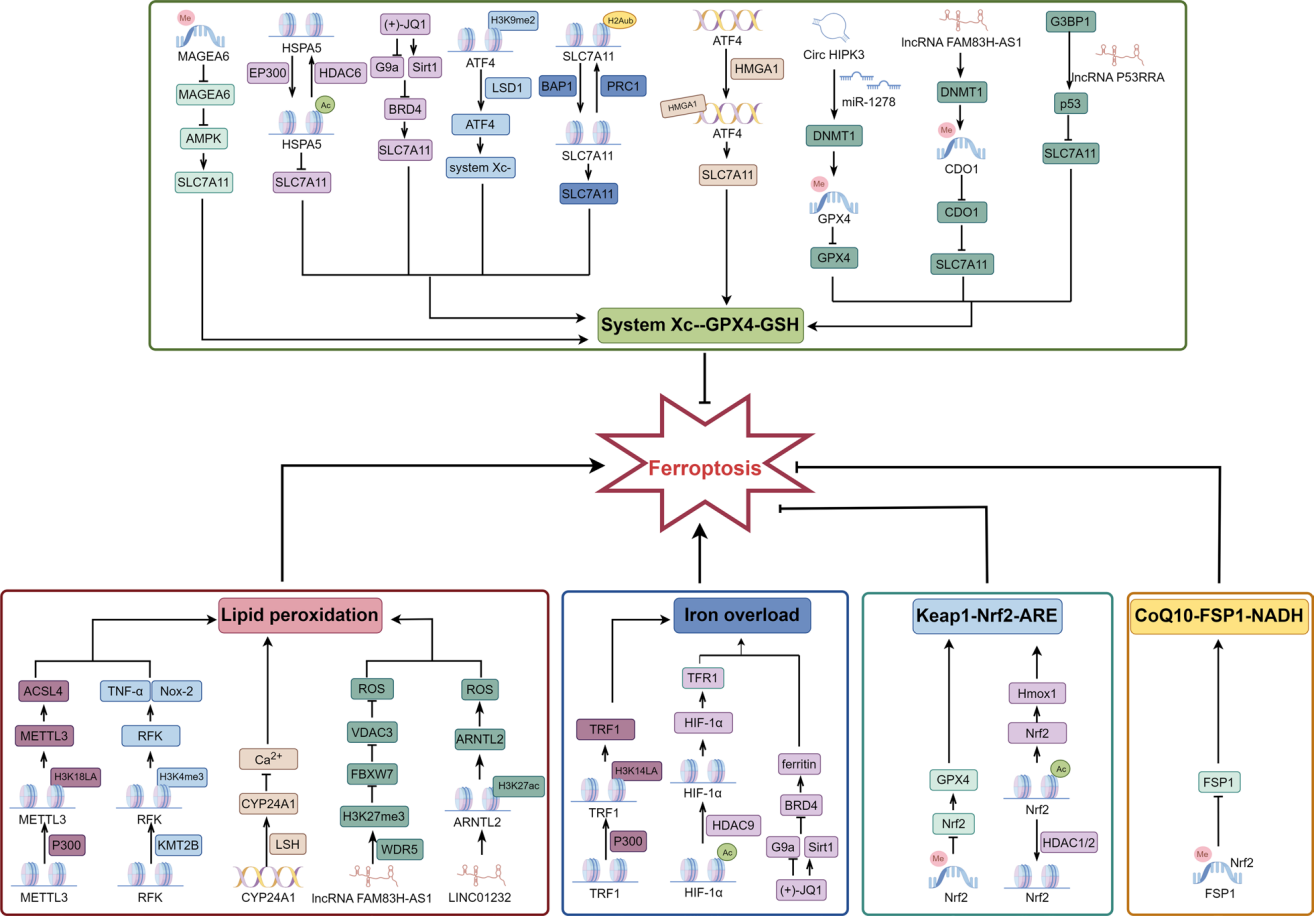
The role of epigenetic regulation in ferroptosis: DNA methylation, histone modification, chromatin remodeling, and ncRNA regulation all play important regulatory roles in the process of ferroptosis, promoting or inhibiting ferroptosis through different pathways. (By Figdraw)

Epigenetic regulation is a mechanism that alters the phenotype of organisms without changing the DNA sequence, primarily including DNA methylation, histone modification, and chromatin remodeling [63]. During sepsis, the transcription of ferroptosis-related genes undergoes significant changes, with epigenetic regulation playing a crucial role in regulating gene transcription, which exerts important regulatory effects on inflammation, microcirculatory dysfunction, and metabolic reprogramming, significantly impacting the occurrence and development of sepsis-associated AKI. Therefore, studying the role of epigenetic regulation in ferroptosis is of great significance for identifying molecular targets for the treatment of sepsis-associated AKI (Fig. 4).

### DNA methylation

DNA methylation refers to the addition of a methyl group to CpG dinucleotides under the action of DNA methyltransferase, thereby altering the chemical properties and structure of DNA and affecting gene expression [64].

Melanoma antigen A6 is a gene that regulates AMPK. Increased expression of melanoma antigen A6 induces AMPK degradation and reduces the expression of SLC7A11, which promotes lipid peroxidation and ferroptosis [65]. Furthermore, AMPK is closely related to metabolic reprogramming, and its degradation may inhibit metabolic reprogramming and exacerbates inflammation, further damaging the kidneys. Hypermethylation of the Nrf2 promoter causes downregulation of Nrf2 expression, inhibiting the binding of Nrf2 to the ARE of GPX4, which downregulates GPX4 and induces ferroptosis. Furthermore, downregulation of Nrf2 expression also exacerbates inflammation and increases tissue damage [66]. Another study on FSP1 suggests that FSP1 promoter contains an Nrf2-binding site overlapping with the CpG island, and DNA methylation of this CpG island prevents FSP1 expression in response to Nrf2 inducers, which silences FSP1 and promotes ferroptosis [67].

In conclusion, DNA methylation plays an important regulatory role in ferroptosis by modulating the expression of ferroptosis-related genes and impacts inflammation and metabolic reprogramming. Therefore, further research on the mechanisms of DNA methylation of ferroptosis-related genes has important clinical significance for the treatment of sepsis-associated AKI.

### Histone modification

Histones are important proteins responsible for maintaining chromatin structure and playing a role in the dynamic and long-term regulation of genes. Histone modifications refer to post-translational modifications of histones, including acetylation, methylation, ubiquitination, and lactylation, which can affect the structure and compactness of chromatin and affect gene expression [68].

Histone acetylation marks are added by histone acetyltransferases, read by bromodomains, and removed by histone deacetylases. E1A binding protein P300 (EP300) acetyltransferase acetylates heat shock protein family A member 5, which promotes ferroptosis by accelerating GPX4 degradation, whereas histone deacetylase 6 can limit heat shock protein family A member 5 acetylation and increase cellular resistance to ferroptosis [69]. Additionally, JQ1 can suppress Bromodomain-containing protein 4 expression by inhibiting the histone methyltransferase G9a or activating the histone deacetylase Sirt1, thereby activating ferritinophagy and downregulating ferroptosis-related genes such as GPX4 and SLC7A11, which promotes ferroptosis [70]. Increased expression of Sirt1 can also block the function of hypoxia inducible factor-1 (HIF-1) and regulate metabolic reprogramming. A study related to Nrf2 indicates that inhibiting histone deacetylase 1/2 can alter the acetylation level of Nrf2 and activate the Nrf2- Heme oxygenase 1 (Hmox1) signaling pathway, thereby resisting ferroptosis [71]. Furthermore, Histone deacetylase 9 can bind to HIF-1, causing deacetylation of HIF-1 and increasing its expression level, which promotes the expression of TFR1 and induces ferroptosis [72].

Histone methylation marks various sites on histone tails and globular domains, balancing their levels through the action of methyltransferases and demethylases [73]. Inhibiting lysine-specific demethylase 1 suppresses the expression of activating transcription factor 4 through histone H3 lysine 9 dimethyl demethylation, which inhibit the system Xc^−^ and promote ferroptosis [74]. In addition, inhibiting lysine-specific demethylase 1 can also enhance the binding of histone H3 lysine 4 dimethyl to the TFR1 promoter sequence, transcriptionally upregulating the expression of TFR1 and ACSL4, and inducing ferroptosis [74]. Lysine-specific methyltransferase 2B is a H3K4 methyltransferase upregulating H3 methylation levels to promote the transcription of the riboflavin kinase gene, which activates the TNF-α/NADPH oxidase 2 axis and promote ferroptosis and inflammation [75].

Ubiquitination refers to the process in which ubiquitin molecules classify intracellular proteins, select target protein molecules, and specifically modify them under the action of a series of specific enzymes. The tumor suppressor BRCA1-associated protein 1 encodes a nuclear deubiquitinase that reduces histone 2A ubiquitination (H2Aub) occupancy on the SLC7A11 promoter, inhibiting SLC7A11 expression, leading to lipid peroxidation, and promoting ferroptosis [76]. However, studies have shown that after the ubiquitin ligase polycomb repressive complex 1 increases H2Aub occupancy on the SLC7A11 promoter, SLC7A11 expression is still inhibited, indicating that the dynamic regulation of H2Aub is important for SLC7A11 inhibition[77].

Histone lysine lactylation was first proposed by Zhang et al. as a novel epigenetic regulatory mechanism in which lactyl groups are added to proteins to regulate their activity and function [78]. During sepsis, cellular metabolic pathways undergo significant alterations, leading to increased lactate production during aerobic glycolysis [79], which can drive the process of lactylation. Studies have shown that lactate can upregulate histone H3 lysine 18 lactylation in the promoter region of METTL3 via G-protein-coupled receptor 81, thereby promoting METTL3 transcription, stabilizing ACSL4 mRNA, and facilitating ferroptosis [80]. Another study also indicated that histone H3 lysine 14 lactylation, derived from lactate accumulation during sepsis, can regulate the transcription of TFR1 and solute carrier family 40 member 1 (SLC40A1), thereby promoting ferroptosis [81].

Histone modifications play an important regulatory role in ferroptosis, affecting iron metabolism, lipid peroxidation, and ferroptosis-related regulatory factors, which have potential regulatory roles in the occurrence of inflammation and metabolic reprogramming, and have significant clinical significance for the treatment of sepsis-associated AKI.

### Chromatin remodeling

Chromatin is a core structure within the interphase nucleus of eukaryotic cells, primarily responsible for storing and regulating genetic information. Its basic structural unit is the nucleosome, which is composed of DNA and histones. Chromatin remodeling is an epigenetic regulatory mechanism that dynamically modulates gene expression by altering the accessibility of chromatin to transcription factors, resulting in changes in the packaging state of chromatin, histones within nucleosomes, and the corresponding DNA molecules [82].

Through bioinformatic analysis of differentially expressed gene datasets related to ferroptosis, gene regulatory networks have revealed that high mobility group AT-hook protein 1 is a crucial transcription factor regulating ferroptosis and is closely associated with it [83]. High mobility group AT-hook protein 1 is a chromatin remodeling protein that promotes the binding of activating transcription factor 4 to the SLC7A11 promoter, thereby enhancing the transcription of SLC7A11 and upregulating its expression, which consequently inhibits ferroptosis [84]. Lymphoid-specific helicase is another chromatin remodeling protein that binds to the promoter of cytochrome P450 family 24 subfamily A member 1, promoting nucleosome eviction and reducing H3K27me3 occupancy, thereby enhancing transcription of cytochrome P450 family 24 subfamily A member 1 and inhibiting excessive intracellular Ca^2+^ influx, which reduces lipid peroxidation and increase resistance to ferroptosis [85].

Chromatin remodeling factors can regulate ferroptosis through chromatin modification and have important connections with inflammation [86], making them potential therapeutic targets for sepsis-associated AKI.

### The regulation of ncRNA

Non-coding RNA (ncRNA) refers to RNA molecules transcribed from the genome that do not encode proteins but play a crucial role in the development and progression of diseases [87]. Based on their structural differences, regulatory ncRNAs are classified into microRNAs (miRNAs), long non-coding RNAs (lncRNAs), circular RNAs (circRNAs), and Piwi-interacting RNAs (piRNAs). Although ncRNAs primarily regulate other genes at the RNA level, recent studies have revealed their critical role in functional regulation at the DNA and chromatin levels [88]. This suggests that ncRNAs may play a pivotal role in the epigenetic regulatory mechanisms of ferroptosis.

Previous studies have shown that ncRNAs can regulate DNA methylation and histone modifications of ferroptosis-related genes. For example, circHIPK3 can bind to miR-1278, preventing its interaction with DNA Methyltransferase 1, thereby promoting the methylation of GPX4 DNA and suppressing its expression, leading to ROS accumulation and promoting ferroptosis [89]. LncRNA FAM83H-AS1 recruits DNA Methyltransferase 1 to increase the methylation level of the CDO1 promoter, leading to the suppression of its expression while upregulating GPX4 and SLC7A11 protein levels, thereby inhibiting ferroptosis [90]. Overexpression of LncRNA BDNF-AS significantly increases H3K27me3 protein enrichment, markedly methylates the CpG island in the FBXW7 promoter region and upregulates voltage-dependent anion channels 3 expression, protecting cells from ferroptosis [91]. LINC01232 interacts with p300, recruits H3K27ac to the promoter region of ARNTL2, enhances the transcriptional activation of ARNTL2, and inhibits erastin-induced ferroptosis [92]. ncRNAs play a dual role in ferroptosis by regulating DNA and histone modifications: on the one hand, they protect genomic integrity to delay ferroptosis; on the other, their dysfunction may cooperatively promote ferroptosis through multiple mechanisms. Future studies should further elucidate the direct targets and dynamic regulatory mechanisms of specific ncRNAs in ferroptosis.

An increasing number of studies have suggested that ncRNAs also play a crucial role in mitotic stability. Research by Lee et al. has demonstrated that lncRNAs are essential for maintaining chromosomal stability in mammalian cells. Specifically, lncRNA NORAD binds to PUMILIO proteins, protecting key factors that maintain chromosomal stability and promoting mitosis, DNA repair, and the expression of DNA replication factors to ensure chromosomal integrity [93]. Another study proposed that lncRNA P53RRA interacts with G3BP1, displacing p53 from the G3BP1 complex and facilitating its nuclear retention, thereby leading to cell cycle arrest, apoptosis, and ferroptosis [94]. Although this study has not directly verified the regulatory role of P53RRA in mitotic stability, its ability to enhance p53 nuclear retention suggests that it may indirectly support mitotic stability by modulating key processes such as cell cycle progression, DNA damage repair, and abnormal cell clearance. Notably, the molecular memory transmission mechanism established by the p53 protein complex during prolonged mitosis has been shown to prevent abnormal cell proliferation, providing theoretical support for the aforementioned hypothesis [95]. Based on this, ncRNAs may influence ferroptosis by modulating mitotic stability, a pivotal mechanism that could serve as a novel therapeutic target for sepsis-associated AKI.

### Clinical feasibility analysis

Clinical research on epigenetic regulation targeting ferroptosis remains in its early stages. A phase I study reported that treatment with the histone deacetylase inhibitor Belinostat significantly upregulated SLC7A11 in nearly all patient samples, suggesting that Belinostat therapy may alleviate ferroptosis in vivo and hold promising clinical potential [96]. Although clinical studies on epigenetic regulation targeting ferroptosis have primarily focused on other diseases, future investigations into its applicability in sepsis-associated AKI are of great clinical significance. While numerous animal studies have demonstrated the therapeutic potential of epigenetic regulation targeting ferroptosis in sepsis-associated AKI, further investigations with larger sample sizes are required to identify more therapeutic targets. With the gradual maturation of therapeutic techniques in animal studies, conducting small-scale clinical trials appropriately can help assess the feasibility and efficacy of new treatments.

## Three-dimensional epigenetics

The basic structural unit of chromatin is the nucleosome composed of DNA and histones. Gene expression is regulated not only by local genetic variations such as DNA methylation and histone modifications but also by the accessibility of chromatin to transcription factors. Chromatin accessibility is closely related to the three-dimensional structure of chromatin, playing an important role in genetic variation which is influenced by the three-dimensional domain structure rather than the linear DNA sequence [97].

It is well known that enhancers do not necessarily regulate the nearest promoter. The interaction between enhancers and promoters typically spans long genomic distances [98]. High-resolution 3D genomic techniques play a crucial role in identifying the connectivity of cis‐elements (promoters, enhancers, silencers, etc.) with transcriptional regulation. The 3D genome, based on chromosome conformation capture (3C) technology, has evolved into more extensive techniques such as chromosome conformation capture‐on‐chip (4C), chromosome conformation capture carbon copy (5C), and high‐throughput chromosome conformation capture (Hi-C), which range from capturing long-range chromatin interactions between two specific genomic loci to performing unbiased interaction analysis across all loci in the entire genome, marking a significant advancement in 3D genomics [99]. Due to the high cost and applicability of 3C technology, new cost-effective FISH probe synthesis methods have sparked significant interest in FISH technology in recent years, further propelling the field of 3D genomics [100].

Recent studies have shown that changes in the three-dimensional structure of chromatin play a regulatory role in ferroptosis. Liu et al. used HiChIP technology to reveal the impact of changes in enhancer-associated three-dimensional chromatin structure on ferroptosis-related gene, which can, respectively, reduce p53 expression and affect ROS production, thereby regulating ferroptosis [101].

Although the mechanisms of three-dimensional epigenetics in ferroptosis are not yet fully understood, due to the importance and complexity of epigenetic regulation in ferroptosis, we believe that future research will uncover more about the three-dimensional epigenetic regulation of ferroptosis, providing more insights into the in-depth mechanisms and clinical implications of ferroptosis.

## Conclusion

During sepsis, inflammation, microcirculatory dysfunction, and metabolic reprogramming cause kidney damage, which may eventually develop into AKI, posing a significant life threat to sepsis patients. Ferroptosis, a form of programmed cell death discovered in recent years, involves iron overload, lipid peroxidation, and multiple regulatory pathways, which plays a crucial role in the occurrence and development of sepsis-associated AKI by influencing inflammation, microcirculatory dysfunction, and metabolic reprogramming, and holds important clinical significance for the treatment of sepsis-associated AKI.

Epigenetic regulation is essential for maintaining homeostasis and is associated with the pathology of various diseases. Increasing evidence suggests that epigenetic regulation plays a critical role in ferroptosis by controlling the expression of genes related to ferroptosis. The main mechanisms of epigenetic regulation include DNA methylation, histone modification, chromatin remodeling, and ncRNA regulation, all of which can affect gene transcription and, thus, regulate ferroptosis. In addition to the individual influence of each regulatory mechanism on ferroptosis, recent studies have shown significant interactive regulatory effects among epigenetic regulation mechanisms. Changes in the three-dimensional structure of chromatin also regulate ferroptosis, which is crucial for discovering new and more effective clinical treatment targets for sepsis-associated AKI.

This review emphasizes the regulatory role of epigenetic regulation in ferroptosis and elucidates the interactive regulatory mechanisms and three-dimensional epigenetics in the process of ferroptosis. However, we still need to maintain skepticism before clinical application. First, the role of epigenetic regulation targeting ferroptosis in sepsis-associated AKI is an emerging field still in its infancy and requires more preclinical research. Although many potential therapeutic factors have been identified, much work remains before treating sepsis-associated AKI by regulating epigenetic targeting of ferroptosis, and inevitable challenges necessitate further clinical studies. Secondly, epigenetic regulation may affect multiple ferroptosis regulatory factors, such as SLC7A11, Nrf2, FSP1, and produce varying effects on ferroptosis, leading to results that may deviate from expectations. Therefore, more extensive and in-depth research on the epigenetic regulation network of ferroptosis is needed. Additionally, relatively mature diagnostic and treatment measures already exist for the clinical management of sepsis-associated AKI [102]. The efficiency, feasibility, and cost-effectiveness of regulating ferroptosis through epigenetic regulation still need to be considered, and personalized treatment should be implemented according to the patient’s specific conditions to provide optimal care. In summary, epigenetic regulation targeting ferroptosis plays an important role in sepsis-associated AKI. Further research on the impact of epigenetic regulation on ferroptosis will ensure a better understanding of the pathogenesis of sepsis-associated AKI and identify new therapeutic targets.

## Data Availability

No datasets were generated or analyzed during the current study.

## Abbreviations

AKI: Acute kidney injury
DAMPs: Damage-associated molecular patterns
PRR: Pattern recognition receptor
TLR: Toll-like receptor
NLRP3: NOD-like receptor protein 3
OXPHOS: Oxidative phosphorylation
AMPK: Adenosine 5 ‘-monophosphate (AMP)-activated protein kinase
TFR1: Transferrin receptor 1
SLC40A1: Solute carrier family 40 member 1
STEAP3: STEAP3 metalloreductase
DMT1: Divalent Metal Transporter 1
CoQ10: Coenzyme Q10
FSP1: Ferroptosis suppressor protein 1
DHFR: Dihydrofolate reductase
BH4: Tetrahydrobiopterin
GCH1: GTP cyclohydrolase 1
PTS: Pyruvoyl tetrahydrobiopterin synthase
SPR: Sepiapterin Reductase
GSH: Glutathione
GSSG: Oxidized glutathione
GPX4: Glutathione peroxidase 4
PUFA: Polyunsaturated fatty acid
LPCAT3: Lysophosphatidylcholine Acyltransferase 3
ACSL4: Acyl-CoA synthetase long-chain family member 4
LOXs: Lipoxygenases
Nrf2: Nuclear factor erythroid-2-related factor 2
Keap1: Kelch-like ECH-associated protein 1
ARE: Antioxidant response element
SLC7A11: Solute carrier family 7 member 11
Hmox1: Heme oxygenase 1
AGER: Advanced glycosylation end-product specific receptor
MAPK: Mitogen-activated protein kinase
MYD88: Myeloid Differentiation Factor 88
TICAM1: Toll Like Receptor Adaptor Molecule 1
8-OHG: 8-Hydroxyguanine
CGAS: Cyclic GMP-AMP synthase
cGAMP: Cyclic GMP-AMP
STING1: Stimulator of interferon response cGAMP interactor 1
CCL2: C-C motif chemokine ligand 2
TGFB1: Transforming growth factor beta 1
AA: Arachidonic acid
CYP450: Cytochrome P450
LOXs: Lipoxygenases
COXs: Cyclooxygenases
HETEs: Hydroxyeicosatetraenoic acids
LDL: Low-density lipoprotein
EP300: E1A binding protein P300
HIF-1: Hypoxia inducible factor-1
H2Aub: Histone 2A ubiquitination
ncRNAs: Non-coding RNAs
miRNA: MicroRNA
lncRNA: Long non-coding RNA
circRNA: Circular RNA
3C: Chromosome Conformation Capture
4C: Chromosome Conformation Capture‐on‐chip
5C: Chromosome Conformation Capture Carbon Copy
Hi-C: High‐throughput Chromosome Conformation Capture
